# Inflammasome Inhibition Prevents Motor Deficit and Cerebellar Degeneration Induced by Chronic Methamphetamine Administration

**DOI:** 10.3389/fnmol.2022.861340

**Published:** 2022-04-01

**Authors:** Jiuyang Ding, Lingyi Shen, Yuanliang Ye, Shanshan Hu, Zheng Ren, Ting Liu, Jialin Dai, Zhu Li, Jiawen Wang, Ya Luo, Qiaojun Zhang, Xiali Zhang, Xiaolan Qi, Jiang Huang

**Affiliations:** ^1^School of Forensic Medicine, Guizhou Medical University, Guiyang, China; ^2^Key Laboratory of Endemic and Ethnic Diseases, Ministry of Education, Guizhou Medical University, Guiyang, China; ^3^School of Basic Medical Science, Guizhou Medical University, Guiyang, China; ^4^Department of Neurosurgery, Liuzhou People’s Hospital, Liuzhou, China; ^5^Good Clinical Practice Center, Affiliated Hospital of Zunyi Medical University, Zunyi, China; ^6^State Key Laboratory of Functions and Applications of Medicinal Plants, Key Laboratory of Pharmaceutics of Guizhou Province, Guizhou Medical University, Guiyang, China

**Keywords:** methamphetamine, inflammasome, cerebellum, degeneration, motor deficit

## Abstract

Methamphetamine (METH), a psychostimulant, has the potential to cause neurodegeneration by targeting the cerebrum and cerebellum. It has been suggested that the NLRP3 inflammasome may be responsible for the neurotoxicity caused by METH. However, the role of NLRP3 in METH-induced cerebellar Purkinje cell (PC) degeneration and the underlying mechanism remain elusive. This study aims to determine the consequences of NLRP3 modulation and the underlying mechanism of chronic METH-induced cerebellar PC degeneration. In METH mice models, increased NLRP3 expression, PC degeneration, myelin sheath destruction, axon degeneration, glial cell activation, and motor coordination impairment were observed. Using the NLRP3 inhibitor MCC950, we found that inhibiting NLRP3 alleviated the above-mentioned motor deficits and cerebellar pathologies. Furthermore, decreased mature IL-1β expression mediated by Caspase 1 in the cerebellum may be associated with the neuroprotective effects of NLRP3 inflammasome inhibition. Collectively, these findings suggest that mature IL-1β secretion mediated by NLRP3-ASC-Caspase 1 may be a critical step in METH-induced cerebellar degeneration and highlight the neuroprotective properties of inflammasome inhibition in cerebellar degeneration.

## Introduction

Methamphetamine (METH), a potent psychostimulant, is widely used throughout the world and has the potential to cause serious injury to the central nervous system, heart and liver (Li et al., 2021). In the central nervous system, METH selectively targets dopaminergic neurons in the substantial nigral area (Ares-Santos et al., 2014; Ding et al., 2020a). METH induces dopaminergic neuron degeneration through oxidative stress, inflammation and endoplasmic reticulum stress, to name a few (Ruan et al., 2020; Kohno et al., 2021). The cerebellum receives substantial signals from different neuronal types including serotoninergic, norepinephrinergic, dopaminergic and acetylcholinergic neurons (Wagner et al., 2021). Evidence suggests that METH could modulate neurotransmitter disorder, which leads to cerebellar dysfunction (Eskandarian Boroujeni et al., 2020). For instance, the serotonin transporter density in METH user cerebellum was lower than that in healthy subject (Sekine et al., 2006).

Methamphetamine may cause cerebellar degeneration, characterized by motor coordination deficits and a decrease in the number of PCs (Boroujeni et al., 2020; Ramshini et al., 2021). However, the precise mechanism by which METH causes cerebellar pathology is unknown.

Inflammasomes, which act as sensors in response to environmental stress insults, are multiprotein complexes (Rathinam and Fitzgerald, 2016; Qiu et al., 2021). Bacterial, viral, and protein aggregates such as neurofibril tangles and β-amyloid, as well as danger-associated molecular patterns such as ATP and oxygen deprivation, all contribute to the formation of the inflammasome (Schroder and Tschopp, 2010; Lamkanfi and Dixit, 2014). Three components comprise the canonical inflammasome complex: the nucleotide-binding domain and leucine-rich repeat-containing protein (NLRP), the apoptosis-associated speck-like protein containing a CARD (ASC), and pro-caspase-1 (Strowig et al., 2012; Nabar and Kehrl, 2019). These three components are referred to as the cytosolic sensor, adaptor protein, and effector, respectively (Song et al., 2017). The NLRP3 inflammasomes may promote the maturation of interleukin-1β (IL-1β) maturation, resulting in inflammation (Martinon et al., 2002). Recent research established that NLRP3 is activated *via* the P53-dependent apoptosis pathway in the hippocampal regions (Du et al., 2019). However, the role of NLRP3 in mice’s cerebellar regions following METH intoxication remains unknown.

Our previous studies have shown that METH could increase α-synuclein (α-syn) and phosphorylated microtubule-associated protein Tau levels in METH mice models (Ding et al., 2020b). Moreover, α-syn could trigger NLRP3 activation in mice Parkinson’s disease model. In addition, inhibiting NLRP3 alleviated α-syn phosphorylation and accumulation in the Parkinson’s disease mice model (Gordon et al., 2018). Nevertheless, whether NLRP3 inhibition could affect α-syn and phosphorylated Tau (p-Tau) aggregation in mice cerebellums after METH administration has not been investigated to this date.

To gain a thorough understanding of the function of NLRP3 in the cerebellar region, we designed a METH-induced mouse model. Besides, the NLRP3 level, motor ability and cerebellar pathology of METH-induced mice were analyzed. Moreover, an NLRP3 inhibitor, MCC950, was utilized to observe the effect of NLRP3 inhibition on motor impairment and cerebellar neurodegeneration in our animal model.

## Materials and Methods

### Animals

C57BL/6J mice (male 20∼24 g, 4∼8 weeks old) were purchased from the Laboratory Animal Center of Guizhou Medical University (Guizhou, China). Mice were kept under a controlled environment with a 12 h light-dark cycle. All mice were housed (four mice per cage) with *ad libitum* access to food and water. All animal experiments were preapproved by the Institutional Animal Care and Use Committee of Guizhou Medical University and were performed according to the National Institutes of Health guide.

### Chronic Methamphetamine Exposure and Experimental Groups

Methamphetamine administration (purity >99%, National Institutes for Food and Drug Control, Guangzhou, China) followed the dosing schedule of Table 1. The chronic METH mouse models were initiated with low doses and concluded with a large challenge dose. The increasing dose and frequency could simulate the progressive use of METH observed in humans and was reported in previous studies (Danaceau et al., 2007).

**TABLE 1:** Dosing schedule of Methamphetamine (METH) treatment (mg/kg).

Day	1	2	3	4	5	6	7	8	9	10	11	12	13	14
8:00	1.0	1.0	1.0	1.0	1.5	1.5	2.0	2.0	2.5	3.0	3.5	4.0	4.5	5.0
10:00				1.0	1.5	1.5	2.0	2.0	2.5	3.0	3.5	4.0	4.5	5.0
12:00				1.0	1.5	1.5	2.0	2.0	2.5	3.0	3.5	4.0	4.5	5.0
14:00		1.0	1.0	1.0	1.5	1.5	2.0	2.0	2.5	3.0	3.5	4.0	4.5	5.0

**FIGURE 1:**
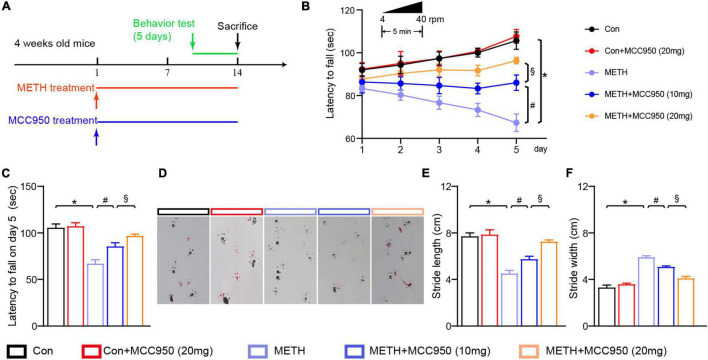
MCC950 ameliorated motor coordination impairment in METH mice model. **(A)** Experimental design of METH and MCC 950 treatment regimen. **(B)** Rotarod test performance in each group mice from day 1 to day 5. **(C)** Latency to fall on day 5 in rotarod test. **(D)** Effect of MCC950 on footprint pattern of mice treated with METH. **(E)** Stride length analysis in footprint test. **(F)** Stride width analysis in footprint test. *n* = 6 per group by one-way ANOVA and Bonferroni’s *post-hoc* analysis, *p < 0.05, compared with the Con group; ^#^*p* < 0.05, compared with the METH-treated group; §*p* < 0.05, compared with the METH + MCC950 (10 mg) group.

The mice were divided into five groups as follows:

Con: Saline was administered intraperitoneally to WT mice, in place of METH;
Con + MCC950 (20 mg): Mice were intraperitoneally injected with MCC950 (20 mg body weight) once daily from day 1 to day 14 as shown in Figure 1A;
Methamphetamine: METH was administered intraperitoneally as shown in Table 1;
Methamphetamine + MCC950 (10 mg): Both METH (Table 1) and MCC950 (10 mg body weight, once daily) were intraperitoneally injected for a total of 14 days;
Methamphetamine + MCC950 (20 mg): Both METH (Table 1) and MCC950 (20 mg body weight, once daily) were intraperitoneally injected for a total of 14 days.

### Rotarod Test

The rotarod tests were carried out to evaluate the body balance and motor coordinative abilities of experimental mice (Nobrega et al., 2013; Zhang C. et al., 2021). The rotarod test was conducted by placing the mice on an accelerating rod. The speed of the rod was set from 4 to 40 rpm. The trial was performed for 5 min. And the test lasted for 5 days (once a day). Animals were trained 3 days before the test. Each animal was tested three times, and an average latency to fall was recorded.

### Footprint Analysis

The footprint analysis were carried out to evaluate the motor coordinative abilities of experimental mice (Nobrega et al., 2013). The testing apparatus is a wooden U-shaped runway that is 10 cm in width and 40 cm in length. A piece of white paper was placed on the bottom of the runway. Before the test, the mice had two forepaws, and two hind paws painted red and black, respectively. Mice were trained for 2 days before the tests. Mice were placed on the beginning of the runway and then allowed to move. The footprints were taken using a camera, and the stride length and width were measured for analysis.

### Western Blot

Cerebellar tissues were homogenized in extraction buffer before centrifugation. The protein concentration in the supernatant was quantified. Then the samples were mixed with the loading buffer before boiling at 99°C for 10 min. The proteins were separated by SDS-PAGE and transferred onto PVDF membranes (Millipore, MA, United States). Targeted protein expression were assessed by using antibodies to rabbit monoclonal α-synuclein antibody (ab138501, 1:1,000 dilution, Abcam, United States), rabbit monoclonal phosphor-Tau antibody (ab32057, 1:800 dilution, Abcam, United States), rabbit monoclonal Tau antibody (ab254256, 1:1,000 dilution, Abcam, United States), rabbit polyclonal NF-200 antibody (18934-1-AP, 1:1,500 dilution, Proteintech, China), rabbit monoclonal MBP antibody (78896, 1:1,000 dilution, CST technology, United States), rabbit monoclonal CNP antibody (5664, 1:1,000 dilution, CST technology, United States), mouse monoclonal GFAP antibody (3670, 1:800 dilution, CST technology, United States), rabbit monoclonal Iba1 antibody (17198, 1:1,000 dilution, CST technology, United States), rabbit polyclonal IL-6 antibody (21865-1-AP, 1:2,000 dilution, Proteintech, China), mouse monoclonal TNF-α antibody (60291-1-Ig, 1:3,000 dilution, Proteintech, China), rabbit monoclonal NLRP3 antibody (ab263899, 1:1,000 dilution, Abcam, United States), rabbit monoclonal ASC antibody (ab155970, 1:5,000 dilution, Abcam, United States), rabbit monoclonal Cleaved Caspase-1 antibody (89332, 1:1,000 dilution, CST technology, United States), rabbit monoclonal Caspase-1 antibody (24232, 1:1,000 dilution, CST technology, United States), rabbit monoclonal Mature IL-1β antibody (A1112, 1:1,000 dilution, ABclonal, China), rabbit monoclonal IL-1β antibody (12507, 1:1,000 dilution, CST technology, United States), mouse monoclonal β-actin antibody (3700, 1:1,000 dilution, CST technology, United States). Membranes were incubated overnight at 4°C with primary antibodies before be blocked in 5% non-fat milk for 1 h. Then the membranes were incubated with adequate secondary antibodies HRP conjugated goat anti-mouse IgG antibody (91196, 1:10,000 dilution, CST technology, United States) or HRP conjugated goat anti-rabbit IgG antibody (ab6721, 1:10,000 dilution, Abcam, United States). Electrochemiluminescence reagents were used to visualize the blot signals. All protein expression levels were normalized to β-actin. Three animals per group were used for western blot analysis.

### Immunohistochemistry and Immunofluorescence Staining

Cerebellar tissues were fixed in 4% PFA for 12 h. For IHC staining, the tissues were embedded in wax. The 3 μm sections were conducted using a microtome (RM2235, Leica, Germany). After antigen recovery and blocking, the sections were incubated with antibodies mouse monoclonal Calbindin antibody (66394-1-Ig, 1:200 dilution, Proteintech, China), rabbit monoclonal α-synuclein antibody (ab138501, 1:200 dilution, Abcam, United States), rabbit monoclonal phosphor-Tau antibody (ab32057, 1:500 dilution, Abcam, United States), rabbit monoclonal CNP antibody (5664, 1:300 dilution, CST technology, United States) and mouse monoclonal GFAP antibody (3670, 1:500 dilution, CST technology, United States) at 4°C overnight. Targeted proteins were visualized using 3, 3 - diaminobenzidine (DAB) kits (CW2069, CWBio, China). Images were acquired using a microscope (CX23, Olympus, Japan). When both the soma and the nuclei appeared, the PC was counted. Three mice per group and three serial sections per mouse were conducted in the experiment.

For immunofluorescence staining, the fixed cerebellar tissues were embedded in optimum cutting temperature compound before sectioning. The 20 μm thickness sections were cut using a microtome (CM 1950, Leica, Germany). Sections were incubated in solution containing 1% Triton X-100 and 5% BSA for 40 min. Then the sections were incubated in primary antibody rabbit monoclonal Iba1 antibody (ab220815, 1:200 dilution, Abcam, MA, United States) at 4°C overnight. Then the Alexa Fluor 488-conjugated secondary antibodies goat anti-rabbit IgG antibody (A-11034, 1:500 dilution, Thermo Fisher Scientific, MA, United States) were incubated for 1 h. Nuclei were stained by mounting Medium (Cat# H-1020, Vector Lab, United States). And when the soma and the nuclei appeared, the glial cell was counted regardless of the projections. Images were captured using a confocal microscope (LSM 780Zeiss, Carl Zeiss, Germany).

### Hematoxylin and Eosin Staining, Luxol Fast Blue Staining and Silver Staining

The 3 μm sections were acquired as described above. Then the sections were dewaxed and rinsed in water. For HE staining, the sections were stained with hematoxylin for 3 min before being rinsed in eosin for 2 min. For LFB staining, the sections were stained with LFB overnight before rinsing in PBS. Then the sections were stained with eosin for 2 min. For silver staining, the sections were rinsed in acid formaldehyde for 5 min, and the sections were then rinsed in 0.25% silver nitrate solution at 37°C for 3 min. After rinsing in gallic hydroxide, the sections were washed using water. All stained sections were dehydrated in gradient alcohol and rinsed in dimethylbenzene before being sealed with gum. Images were acquired using a microscope (CX23, Olympus, Japan). Three mice per group and three serial sections per mouse were conducted in each experiment.

### Statistical Analysis

All data were expressed as mean ± standard deviation. All analyses were analyzed using SPSS 19.0, and charts were conducted using Graphpad prism 9.2. The one-way ANOVA with Bonferroni’s multiple comparison *post-hoc* test was conducted for the statistical analysis. Randomization and blind analyses were used in behavioral test and pathological analysis. Statistical significance was set at *p* < 0.05. The number of different experimental groups is reported in the figure legends.

## Results

### Restoration of Motor Performance in Chronic Methamphetamine Mice Treated With NLRP3 Inhibitor MCC950

The rotarod test was used to investigate motor deficits in mice following METH intoxication and determine whether the motor deficit was restored when the selective NLRP3 inhibitor MCC950 was used. The rotarod test revealed that the latency to fall was significantly reduced in METH-treated mice compared to control mice (Figure 1B). However, administration of MCC950 (10 and 20 mg/kg) could reverse the decline in fall latency of METH-induced mice (Figure 1B). 20 mg/kg MCC950 normalized the fall latency in mice treated with METH when compared to control mice on day 5 (Figure 1C). METH-treated mice had a shorter stride length and a wider stride width during the gait test compared to the control mice. In contrast, the MCC950 intervention could reverse the effect of METH *in vivo* (Figures 1D–F).

### Administration of MCC950 Protected Against Purkinje Cell Soma Loss in Methamphetamine Mice Model

**FIGURE 2:**
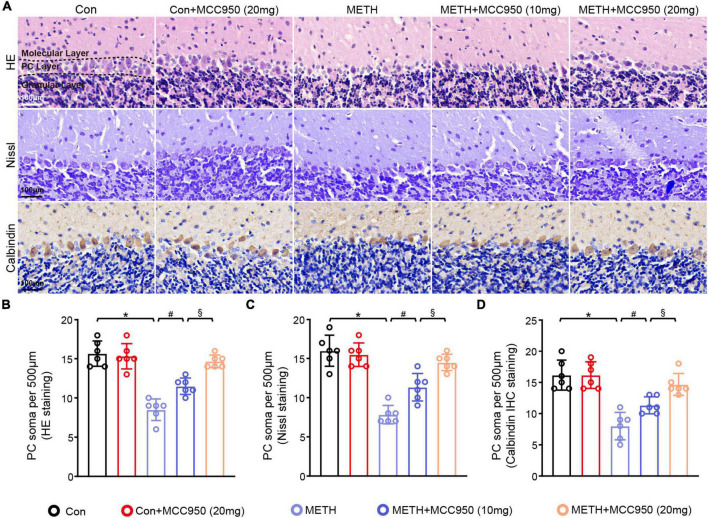
MCC950 alleviated PC soma number loss in chronic METH mice model. **(A)** HE staining, Nissl staining and Calbindin IHC staining in the cerebellar area. **(B)** Comparison of PC soma number from HE staining results. **(C)** Comparison of PC soma number from Nissl staining results. **(D)** Comparison of PC soma number from Calbindin IHC staining results. *n* = 6 per group by one-way ANOVA and Bonferroni’s *post-hoc* analysis, *p < 0.05, compared with the Con group; ^#^*p* < 0.05, compared with the METH-treated group; §*p* < 0.05, compared with the METH + MCC950 (10 mg) group.

Next we investigated the mechanism of action of the NLRP3 inhibitor MCC950 in METH-induced PC degeneration. HE staining, nissl staining, Calbindin (A PC marker) and IHC staining were used to assess the number of PC soma in cerebellar regions. The number of PC soma was smaller in the METH mice group than in the control group. However, MCC950 at 10 or 20 mg/kg could alleviate the loss of PC soma number caused by METH. In this regard, no significant difference in the PC soma number was found between the Con + MCC950 (20 mg/kg) group and control mice (Figures 2A–D).

### Treatment With MCC950 Reduced α-Synuclein and Phosphorylated Tau Accumulation in the Cerebellar Purkinje Cell

**FIGURE 3:**
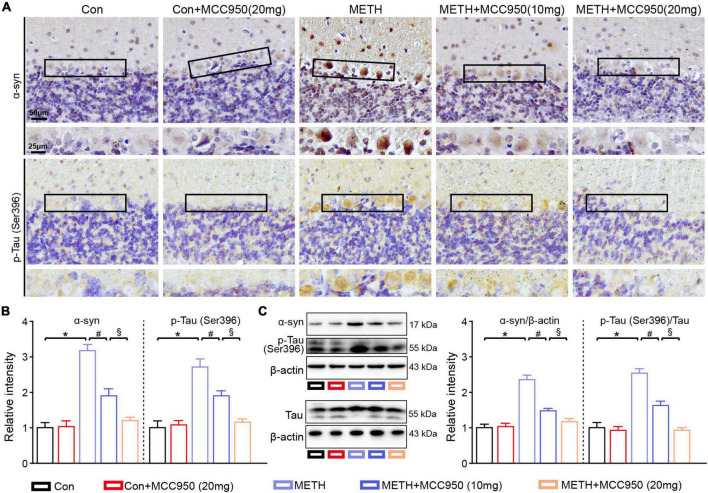
MCC950 reversed α-syn and p-Tau level increasing. **(A)** Representative images of α-syn and pSer396-Tau IHC staining. **(B)** Relative intensity of α-syn and pSer396-Tau in cerebellar areas. **(C)** Western blot and quantification for α-syn and pSer396-Tau in cerebellum. *n* = 6 per group by one-way ANOVA and Bonferroni’s *post-hoc* analysis, *p < 0.05, compared with the Con group; ^#^*p* < 0.05, compared with the METH-treated group; §*p* < 0.05, compared with the METH + MCC950 (10 mg) group.

Next, we assessed whether NLRP3 inhibition altered α-syn and p-Tau levels within the cerebellar regions by α-syn and p-Tau IHC staining. We found that α-syn and p-Tau mainly accumulated in cerebellar PC soma after METH treatment from the high magnification images (Magnified black box in the lower panel). A decline in α-syn and p-Tau accumulation was found in the cerebellum of METH-treated mice after administration of MCC950. Moreover, the α-syn and p-Tau levels in the METH + MCC950 (20 mg/kg) group mice were lower than in METH + MCC950 (10 mg/kg) group mice (Figures 3A,B). Consistently, the protein levels of GFAP and Iba 1 were elevated in the METH group compared to the control group and reversed by MCC950 administration (Figure 3C).

### Pharmacological Inhibition With MCC950 Prevents Axonal Degeneration and Myelin Sheath Destruction in the Cerebellum

**FIGURE 4:**
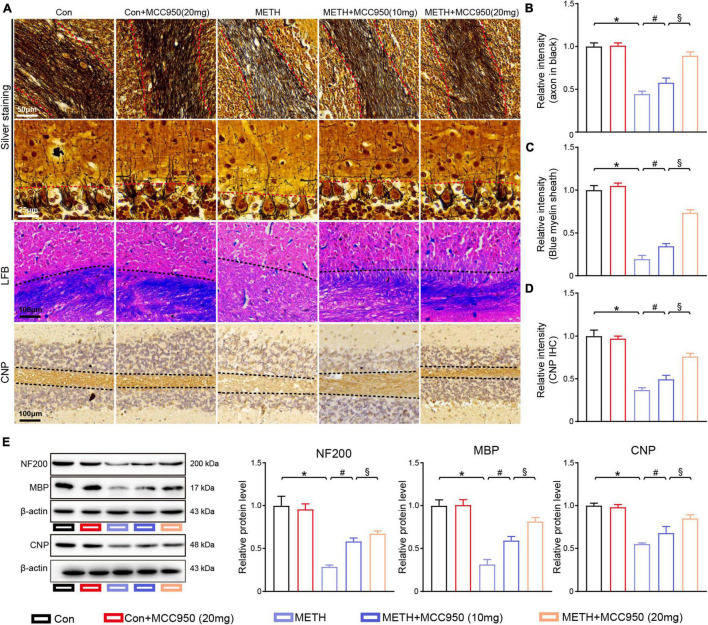
MCC950 diminished the axon degeneration and myelin loss in cerebellum. **(A)** Representative images of silver staining, LFB staining, CNP IHC staining in cerebellar areas. **(B)** Intensity of axons analysis. **(C)** Myelin sheath intensity analysis. **(D)** Relative intensity of CNP in cerebellar areas. **(E)** Western blot and quantification for NF-200, MBP, and CNP in cerebellum. *n* = 6 per group by one-way ANOVA and Bonferroni’s *post-hoc* analysis, *p < 0.05, compared with the Con group; ^#^*p* < 0.05, compared with the METH-treated group; §*p* < 0.05, compared with the METH + MCC950 (10 mg) group.

To characterize cerebellar axonal degeneration after METH-induced injury, we conducted silver staining to examine the axons in the cerebellar white matter. Silver staining showed a decline in axon intensities in the white matter tracts of the METH mice, whereas both 10 and 20 mg/kg MCC950 treatment preserved axonal intensity assessed after METH intoxication (Figures 4A,B). Immunofluorescence of NF200 (an axon-specific protein) showed axonal degeneration in METH group mice. Consistent with the silver staining results, MCC950 attenuated the reduction in NF200 levels in the cerebellum of mice treated with METH (Figure 4E).

To assess the protective effect of MCC950 against METH-induced myelin loss, we conducted LFB staining and CNP immunostaining. Prophylactic treatment with MCC950 protected the myelin sheath loss in the cerebellum of METH-treated mice (Figures 4A,C,D). Consistent with the results of the staining sections, myelin-specific protein CNP and MBP immunoblotting showed that inflammasome modulation with MCC950 reversed the myelin-specific proteins loss induced by METH (Figure 4E).

### MCC950 Attenuated Glial Cell Activation in Methamphetamine Mice Model

**FIGURE 5:**
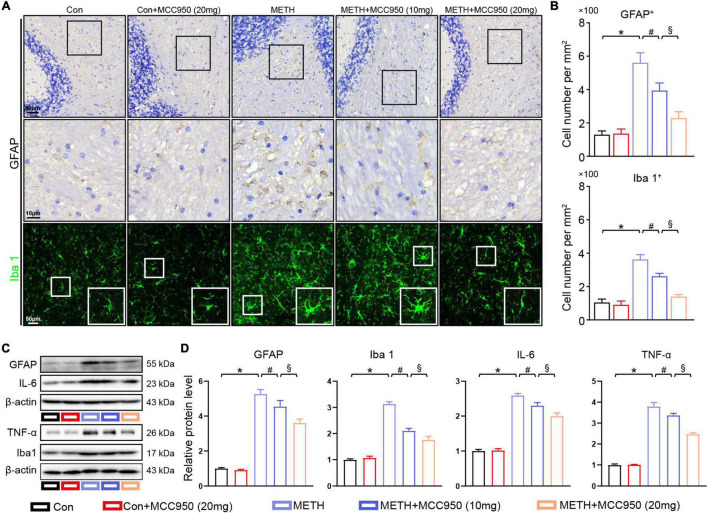
MCC950 alleviated glial activation in cerebellar regions. **(A)** Representative images of GFAP IHC staining and Iba 1 IF staining in cerebellar regions. **(B)** Quantification of GFAP and Iba 1 positive cell number in cerebellar subregions. **(C,D)** Western blot and quantification for GFAP, Iba 1, IL-6, and TNF-α in cerebellum. *n* = 6 per group by one-way ANOVA and Bonferroni’s *post-hoc* analysis, *p < 0.05, compared with the Con group; ^#^*p* < 0.05, compared with the METH-treated group; §*p* < 0.05, compared with the METH + MCC950 (10 mg) group.

To investigate the effect of MCC950 on glial activation induced by METH, we employed GFAP and Iba 1 immunostaining to quantify the number of astrocytes and microglia. We found that METH increased the numbers of astrocytes and microglia within the cerebellum. METH-treated mice that received MCC950 exhibited significantly fewer astrocytes and microglia cells in the cerebellum than control mice (Figures 5A,B). Immunoblotting showed a significant increase in GFAP and Iba 1 levels in METH-treated mice compared with control mice. Inhibition of NLRP3 by MCC950 enhanced IL-6 and TNF-α upregulation in the cerebellum of METH-treated mice (Figures 5C,D).

### Modulating NLRP3 by MCC950 Decreased Interleukin-1β Secretion Through Caspase-1 Dependent Pathway in Methamphetamine Mice Model

**FIGURE 6:**
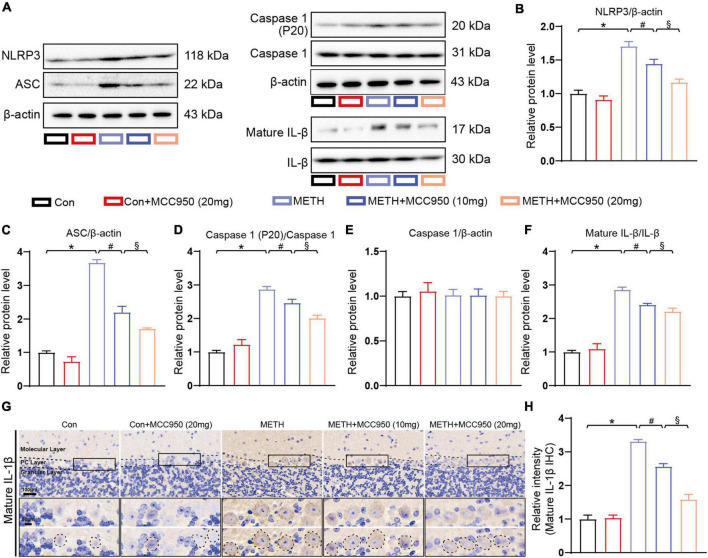
Effect of MCC950 on NLRP3 pathway in METH mice model. **(A)** Representative western blot bands of NLRP3, ASC, Caspase-1, Cleaved Caspase-1, mature IL-1β, and IL-1β of mice cerebellar tissues in each group. **(B–F)** Quantification of NLRP3, ASC, Caspase-1, Cleaved Caspase-1, mature IL-1β, and IL-1β protein levels of mice cerebellar tissues from western blot bands. *n* = 3 per group. **(G)** Representative images of mature IL-1β IHC staining in cerebellar areas. **(H)** Relative intensity of mature IL-1β in cerebellar areas. One-way ANOVA and Bonferroni’s *post-hoc* analysis, *p < 0.05, compared with the Con group; ^#^*p* < 0.05, compared with the METH-treated group; §*p* < 0.05, compared with the METH + MCC950 (10 mg) group.

To explore the mechanism underlying the protective effect of MCC950 in METH-induced cerebellar degeneration, we conducted an immunoblotting analysis of NLRP3 pathway proteins including NLRP3, ASC, Caspase-1, Cleaved Caspase-1 (P20), IL-1β and mature IL-1β. We found that NLRP3, ASC, Cleaved Caspase-1 and mature IL-1β protein expression were elevated in the cerebellum of METH-treated mice. Furthermore, MCC950 (at 10 and 20 mg/kg) suppressed METH-induced increases in protein levels of NLRP3, Cleaved Caspase-1 and mature IL-1β. Moreover, the cerebellar NLRP3, Cleaved Caspase-1 and mature IL-1β protein levels were significantly lower in the (METH + 20 mg/kg MCC950) group than in the (METH + 10 mg/kg MCC950) group (Figures 6A–F). Finally, IHC staining revealed that mature IL-1β intensity was significantly decreased in the (METH + MCC950) group mice compared to METH-treated mice (Figures 6G,H).

## Discussion

It has been established that the nigrostriatal dopaminergic system is vulnerable to METH exposure since METH can be transferred into the cytoplasm by dopamine transporters (Huang et al., 2020). A recent study indicated that METH might lead to electrophysiological and morphological alternations of cerebellar PC (Ramshini et al., 2021). In the present study, we demonstrated that chronic METH could influence behavioral performance and cerebellar neurodegeneration involving a decrease in the number of PC, α-syn and p-Tau accumulation, axon degeneration, myelin sheath destruction and glial activation.

Importantly, the NLRP3-ASC-Caspase 1 pathway is activated during this process. To the best of our knowledge, this is the first study to expound that modulation of NLRP3 by MCC950 exerts a protective effect against motor deficits and cerebellar degeneration in METH-treated mice. These results suggested that NLRP3 might be a therapeutic target, and the NLRP3 pathway is associated with METH-induced cerebellar degeneration.

An increasing body of evidence suggests that NLRP3 participates in the formation of inflammasomes in several neurodegenerative diseases, such as Alzheimer’s disease and Parkinson’s disease (Yang et al., 2020). Moreover, targeting NLRP3 can alleviate learning and memory deficits in an Alzheimer’s disease mice model (Lonnemann et al., 2020). To confirm that NLRP3 inhibition alleviates the behavioral impairment induced by METH, we conducted behavioral tests. Indeed, we found NLRP3 inhibition exerts protective effects on motor balance and coordination impairments induced by METH (Figures 1B–F). Importantly, we found that 20 mg/kg MCC950 was more effective than 10 mg/kg MCC950 in response to METH-induced behavior impairment.

It has been established that the PC is a unique kind of neuron that represents the only efferent neuron in the cerebellar cortex, essential for cerebellar function (Schonewille et al., 2021). It is well documented that the PC number is reduced in response to METH (Ramshini et al., 2021). Consistent with this study, we found a decline in PC number in mice cerebellum after METH intoxication, which could be alleviated by 10 and 20 mg/kg of MCC950. Previous studies showed the PC loss was sufficient to drive behavioral chanes such as the impaired coordination of limb movement (Zhang H. et al., 2021). Since cerebellar PC dysfunction or degeneration is frequently accompanied with cerebellar ataxia (Aoki et al., 2022). In our study, we showed a reduction of PC number in METH group mice, which was sufficient to drive motor coordination deficit in rotarod test and footprint analysis test.

The accumulation of α-syn and p-Tau are the hallmarks of Parkinson’s disease and Alzheimer’s disease, respectively (Vasili et al., 2019; Yin et al., 2021). It is widely acknowledged that α-syn and p-Tau are “teammates” in neurodegenerative disease (Moussaud et al., 2014). Our previous studies revealed that both α-syn and p-Tau are upregulated in several brain regions in response to METH (Ding et al., 2020b). The present study found increased cerebellar levels of α-syn and p-Tau in METH-treated mice, especially in the PCs (Figure 3). Furthermore, we found that MCC950 could restore cerebellar α-syn and p-Tau aggregation levels in chronic METH-treated mice. These findings suggested that therapeutic NLRP3 inhibition could alleviate pathological α-syn and p-Tau accumulations in METH-treated mice, consistent with findings of a recent study that showed that pharmacological inhibition of NLRP3 could protect against α-syn pathology in a Parkinson’s disease mice model (Gordon et al., 2018). Importantly, α-syn can trigger NLRP3 activation in Parkinson’s disease, leading to α-syn accumulation (Codolo et al., 2013; Zhang C. et al., 2021). We hypothesize that α-syn accumulation and NLRP3 activation form a vicious cycle, and disrupting this cycle by NLRP3 inhibition could alleviate α-syn pathologies in METH-treated mice.

The PC axons are the sole efferent neuron fiber in the cerebellum and are crucial to PC function (Falcon-Moya et al., 2018). Besides, the myelin sheaths, formed by oligodendrocytes, are vital for PC signal output (Bechet et al., 2020). In the present study, we observed the cerebellar axon and myelin sheath morphology in METH-treated mice. Silver and LFB stainings showed reduced axon intensity, myelin sheath destruction, respectively, and pharmacological inhibition of NLRP3 alleviated axon and myelin sheath pathologies induced by METH (Figure 4).

In addition to PC degeneration, glial cells activation participates in the neurodegenerative diseases process (Saijo et al., 2009). It has been suggested that activated glial cells act as phagocytic cells, which can clear the abnormal protein aggregates and cell debris and act as inflammatory factors secreting cells (Chen et al., 2021). Our previous study showed that METH triggered glial cell activation in the hippocampal and substantial nigral region (Ding et al., 2020a). Accordingly, we assessed whether METH would affect glial cell activation in the cerebellar region. The immunolabeling of GFAP (an astrocyte marker) and Iba 1 (a microglia marker) in mice cerebellar sections revealed a significant increase in astrocyte and microglia number and inflammatory factors after 2 weeks of METH treatment. However, glial activation was suppressed in METH-treated mice after administration of MCC950, which was supported by reduced inflammatory factors such as TNF-α and IL-6 (Figure 5). Given that inflammatory factors may trigger neuronal dysfunction, we hypothesize that NLRP3 inhibition can alleviate cerebellar degeneration *via* modulation of inflammation.

To explore the mechanisms involved in the METH-induced cerebellar degeneration, we quantified NLRP3-ASC-Caspase 1-IL-1β pathway protein levels. Treatment with METH triggered the NLRP3 sensor, which led to the upregulation of downstream proteins including ASC, Caspase 1 and mature IL-1β. Furthermore, pharmacological inhibition of NLRP3 suppressed activation of the NLRP3-ASC-Caspase 1-IL-1β pathway. Given that mature IL-1β secretion has been shown to be a key mediator in inflammation-induced neuronal apoptosis in neurodegenerative disease (Rui et al., 2021), our mechanistic finding suggests that pharmacological inhibition of NLRP3 may alleviate inflammation through Caspase 1 dependent mature IL-1β secretion (Figure 6). Moreover, we found mature IL-1β accumulation in the cytoplasm of PC in METH-treated mice, indicating that glial-derived mature IL-1β was transferred into the PCs. In addition, the transfer could be blocked by MCC950 (Figures 6G,H). It has been reported that NLRP3 dependent mature IL-1β secretion can induce neurodegeneration in several mice models (Holbrook et al., 2021), consistent with our findings.

**FIGURE 7:**
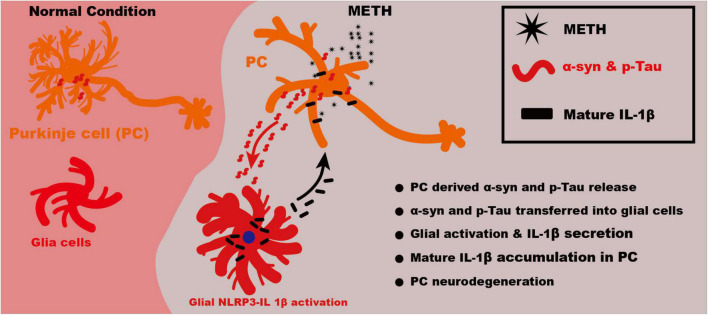
Schematic illustration of mechanism of NLRP3–IL-1β mediating METH induced cerebellar neurodegeneration. Upon METH treatment, the α-syn and p-Tau level was increased in PCs. The increased α-syn and p-Tau might release from neurons and be uptaken by glia cells. The activated glia could secrete inflammatory factors IL-1β through NLRP3 activation. The excess IL-1β damage the neurons, which lead to α-syn and p-Tau level increasing. Thus the neuronal α-syn and p-Tau accumulation and microglial NLRP3 activation form a vicious cycle.

Taking together, our study showed that METH could upregulate the α-syn and p-Tau level in PCs. The increased α-syn and p-Tau might release from PCs and be uptaken by glial cells. The activated glial cells could secrete inflammatory factors IL-1β through NLRP3 activation. The excess IL-1β damage the neurons by inhibiting neuronal autophagy flux, which lead to α-syn and p-Tau level increasing. Thus the neuronal α-syn and p-Tau accumulation and microglial NLRP3 activation form a vicious cycle (Figure 7).

Overall, we demonstrated that METH could cause cerebellar neurodegeneration and motor coordination deficit through NLRP3 dependent mature IL-1β secretion-induced inflammation in METH-treated mice. Moreover, NLRP3 inhibition alleviated cerebellar pathologies and behavior abnormalities. Our study provided compelling evidence of the therapeutic effect of NLRP3 inhibitor in treating METH-induced cerebellar neurodegeneration. Nonetheless, further studies are warranted to better understand other inflammatory pathways involved in METH-induced cerebellar degeneration.

## Data Availability Statement

The original contributions presented in the study are included in the article/supplementary material, further inquiries can be directed to the corresponding author.

## Ethics Statement

The animal study was reviewed and approved by the Institutional Animal Care and Use Committee of Guizhou Medical University.

## Author Contributions

JYD and JH conceived and designed the research. JYD, LS, YY, SH, ZR, JLD, ZL, JW, YL, QZ, and XZ performed the experiments. ZR, XQ, and TL contributed reagents, materials, analysis tools. JYD and LS wrote the manuscript. All authors edited and approved of the final manuscript.

## Conflict of Interest

The authors declare that the research was conducted in the absence of any commercial or financial relationships that could be construed as a potential conflict of interest.

## Publisher’s Note

All claims expressed in this article are solely those of the authors and do not necessarily represent those of their affiliated organizations, or those of the publisher, the editors and the reviewers. Any product that may be evaluated in this article, or claim that may be made by its manufacturer, is not guaranteed or endorsed by the publisher.

## References

[B1] Aoki H. Higashi M. Okita M. Ando N. Murayama S. Ishikawa K. (2022). Thymidine kinase 2 and mitochondrial protein COX I in the cerebellum of patients with spinocerebellar ataxia type 31 caused by penta-nucleotide repeats (TTCCA) n. *Cerebellum.* 10.1007/s12311-021-01364-2 35084690 PMC9883315

[B2] Ares-Santos S. Granado N. Espadas I. Martinez-Murillo R. Moratalla R. (2014). Methamphetamine causes degeneration of dopamine cell bodies and terminals of the nigrostriatal pathway evidenced by silver staining. *Neuropsychopharmacology* 39 1066–1080. 10.1038/npp.2013.307 24169803 PMC3957101

[B3] Bechet S. O’sullivan S. A. Yssel J. Fagan S. G. Dev K. K. (2020). Fingolimod rescues demyelination in a mouse model of Krabbe’s disease. *J. Neurosci.* 40 3104–3118. 10.1523/JNEUROSCI.2346-19.2020 32127495 PMC7141882

[B4] Boroujeni M. E. Nasrollahi A. Boroujeni P. B. Fadaeifathabadi F. Farhadieh M. Tehrani A. M. (2020). Exposure to methamphetamine exacerbates motor activities and alters circular RNA profile of cerebellum. *J. Pharmacol. Sci.* 144 1–8. 10.1016/j.jphs.2020.05.010 32576439

[B5] Chen M. Lai X. Wang X. Ying J. Zhang L. Zhou B. (2021). Long Non-coding RNAs and circular RNAs: insights into microglia and astrocyte mediated neurological diseases. *Front. Mol. Neurosci.* 14:745066. 10.3389/fnmol.2021.745066 34675776 PMC8523841

[B6] Codolo G. Plotegher N. Pozzobon T. Brucale M. Tessari I. Bubacco L. (2013). Triggering of inflammasome by aggregated alpha-synuclein, an inflammatory response in synucleinopathies. *PLoS One* 8:e55375. 10.1371/journal.pone.0055375 23383169 PMC3561263

[B7] Danaceau J. P. Deering C. E. Day J. E. Smeal S. J. Johnson-Davis K. L. Fleckenstein A. E. (2007). Persistence of tolerance to methamphetamine-induced monoamine deficits. *Eur. J. Pharmacol.* 559 46–54. 10.1016/j.ejphar.2006.11.045 17239369

[B8] Ding J. Hu S. Meng Y. Li C. Huang J. He Y. (2020a). Alpha-synuclein deficiency ameliorates chronic methamphetamine induced neurodegeneration in mice. *Toxicology* 438 152461. 10.1016/j.tox.2020.152461 32278788

[B9] Ding J. Lian Y. Meng Y. He Y. Fan H. Li C. (2020b). The effect of alpha-synuclein and Tau in methamphetamine induced neurotoxicity *in vivo* and *in vitro*. *Toxicol. Lett.* 319 213–224. 10.1016/j.toxlet.2019.11.028 31783120

[B10] Du L. Shen K. Bai Y. Chao J. Hu G. Zhang Y. (2019). Involvement of NLRP3 inflammasome in methamphetamine-induced microglial activation through miR-143/PUMA axis. *Toxicol. Lett.* 301 53–63. 10.1016/j.toxlet.2018.10.020 30394308

[B11] Eskandarian Boroujeni M. Peirouvi T. Shaerzadeh F. Ahmadiani A. Abdollahifar M. A. Aliaghaei A. (2020). Differential gene expression and stereological analyses of the cerebellum following methamphetamine exposure. *Addict. Biol.* 25:e12707. 10.1111/adb.12707 30714656

[B12] Falcon-Moya R. Losada-Ruiz P. Sihra T. S. Rodriguez-Moreno A. (2018). Cerebellar kainate receptor-mediated facilitation of glutamate release requires Ca(2+)-calmodulin and PKA. *Front. Mol. Neurosci.* 11:195. 10.3389/fnmol.2018.00195 29928192 PMC5997777

[B13] Gordon R. Albornoz E. A. Christie D. C. Langley M. R. Kumar V. Mantovani S. (2018). Inflammasome inhibition prevents alpha-synuclein pathology and dopaminergic neurodegeneration in mice. *Sci. Transl. Med.* 10:eaah4066. 10.1126/scitranslmed.aah4066 30381407 PMC6483075

[B14] Holbrook J. A. Jarosz-Griffiths H. H. Caseley E. Lara-Reyna S. Poulter J. A. Williams-Gray C. H. (2021). Neurodegenerative disease and the NLRP3 inflammasome. *Front. Pharmacol.* 12:643254. 10.3389/fphar.2021.643254 33776778 PMC7987926

[B15] Huang J. Yang G. Li Z. Leung C. K. Wang W. Li Y. (2020). Involvement of dopamine D3 receptor and dopamine transporter in methamphetamine-induced behavioral sensitization in tree shrews. *Brain Behav.* 10:e01533. 10.1002/brb3.1533 31943832 PMC7010569

[B16] Kohno M. Dennis L. E. Mccready H. Hoffman W. F. (2021). Dopamine dysfunction in stimulant use disorders: mechanistic comparisons and implications for treatment. *Mol. Psychiatry.* 10.1038/s41380-021-01180-4 34117366 PMC8664889

[B17] Lamkanfi M. Dixit V. M. (2014). Mechanisms and functions of inflammasomes. *Cell* 157 1013–1022. 10.1016/j.cell.2014.04.007 24855941

[B18] Li J. H. Liu J. L. Zhang K. K. Chen L. J. Xu J. T. Xie X. L. (2021). The adverse effects of prenatal METH exposure on the offspring: a review. *Front. Pharmacol.* 12:715176. 10.3389/fphar.2021.715176 34335277 PMC8317262

[B19] Lonnemann N. Hosseini S. Marchetti C. Skouras D. B. Stefanoni D. D’Alessandro A. (2020). The NLRP3 inflammasome inhibitor OLT1177 rescues cognitive impairment in a mouse model of Alzheimer’s disease. *Proc. Natl. Acad. Sci. U.S.A.* 117 32145–32154. 10.1073/pnas.2009680117 33257576 PMC7749353

[B20] Martinon F. Burns K. Tschopp J. (2002). The inflammasome: a molecular platform triggering activation of inflammatory caspases and processing of proIL-beta. *Mol. Cell* 10 417–426. 10.1016/s1097-2765(02)00599-3 12191486

[B21] Moussaud S. Jones D. R. Moussaud-Lamodiere E. L. Delenclos M. Ross O. A. Mclean P. J. (2014). Alpha-synuclein and tau: teammates in neurodegeneration? *Mol. Neurodegener.* 9:43. 10.1186/1750-1326-9-43 25352339 PMC4230508

[B22] Nabar N. R. Kehrl J. H. (2019). Inflammasome inhibition links IRGM to innate immunity. *Mol. Cell* 73 391–392. 10.1016/j.molcel.2019.01.029 30735651

[B23] Nobrega C. Nascimento-Ferreira I. Onofre I. Albuquerque D. Conceicao M. Deglon N. (2013). Overexpression of mutant ataxin-3 in mouse cerebellum induces ataxia and cerebellar neuropathology. *Cerebellum* 12 441–455. 10.1007/s12311-012-0432-0 23242710

[B24] Qiu X. Wang Q. Hou L. Zhang C. Wang Q. Zhao X. (2021). Inhibition of NLRP3 inflammasome by glibenclamide attenuated dopaminergic neurodegeneration and motor deficits in paraquat and maneb-induced mouse Parkinson’s disease model. *Toxicol. Lett.* 349 1–11. 10.1016/j.toxlet.2021.05.008 34052309

[B25] Ramshini E. Sheykhzade M. Dabiri S. Shabani M. (2021). Cannabinoid CB1 receptor mediates METH-induced electrophysiological and morphological alterations in cerebellum Purkinje cells. *Hum. Exp. Toxicol.* 40 940–951. 10.1177/0960327120975448 33249856

[B26] Rathinam V. A. Fitzgerald K. A. (2016). Inflammasome complexes: emerging mechanisms and effector functions. *Cell* 165 792–800. 10.1016/j.cell.2016.03.046 27153493 PMC5503689

[B27] Ruan Q. T. Yazdani N. Blum B. C. Beierle J. A. Lin W. Coelho M. A. (2020). A mutation in Hnrnph1 that decreases methamphetamine-induced reinforcement, reward, and dopamine release and increases synaptosomal hnRNP H and mitochondrial proteins. *J. Neurosci.* 40 107–130. 10.1523/JNEUROSCI.1808-19.2019 31704785 PMC6939476

[B28] Rui W. Xiao H. Fan Y. Ma Z. Xiao M. Li S. (2021). Systemic inflammasome activation and pyroptosis associate with the progression of amnestic mild cognitive impairment and Alzheimer’s disease. *J. Neuroinflammation* 18:280. 10.1186/s12974-021-02329-2 34856990 PMC8638109

[B29] Saijo K. Winner B. Carson C. T. Collier J. G. Boyer L. Rosenfeld M. G. (2009). A Nurr1/CoREST pathway in microglia and astrocytes protects dopaminergic neurons from inflammation-induced death. *Cell* 137 47–59. 10.1016/j.cell.2009.01.038 19345186 PMC2754279

[B30] Schonewille M. Girasole A. E. Rostaing P. Mailhes-Hamon C. Ayon A. Nelson A. B. (2021). NMDARs in granule cells contribute to parallel fiber-Purkinje cell synaptic plasticity and motor learning. *Proc. Natl. Acad. Sci. U.S.A.* 118:e2102635118. 10.1073/pnas.2102635118 34507990 PMC8449340

[B31] Schroder K. Tschopp J. (2010). The inflammasomes. *Cell* 140 821–832. 20303873 10.1016/j.cell.2010.01.040

[B32] Sekine Y. Ouchi Y. Takei N. Yoshikawa E. Nakamura K. Futatsubashi M. (2006). Brain serotonin transporter density and aggression in abstinent methamphetamine abusers. *Arch. Gen. Psychiatry* 63 90–100. 10.1001/archpsyc.63.1.90 16389202

[B33] Song L. Pei L. Yao S. Wu Y. Shang Y. (2017). NLRP3 inflammasome in neurological diseases, from functions to therapies. *Front. Cell. Neurosci.* 11:63. 10.3389/fncel.2017.00063 28337127 PMC5343070

[B34] Strowig T. Henao-Mejia J. Elinav E. Flavell R. (2012). Inflammasomes in health and disease. *Nature* 481 278–286. 10.1038/nature10759 22258606

[B35] Vasili E. Dominguez-Meijide A. Outeiro T. F. (2019). Spreading of alpha-Synuclein and Tau: a systematic comparison of the mechanisms involved. *Front. Mol. Neurosci.* 12:107. 10.3389/fnmol.2019.00107 31105524 PMC6494944

[B36] Wagner M. J. Savall J. Hernandez O. Mel G. Inan H. Rumyantsev O. (2021). A neural circuit state change underlying skilled movements. *Cell* 184 3731–3747.e21. 10.1016/j.cell.2021.06.001 34214470 PMC8844704

[B37] Yang J. Wise L. Fukuchi K. I. (2020). TLR4 cross-Talk With NLRP3 inflammasome and complement signaling pathways in Alzheimer’s disease. *Front. Immunol.* 11:724. 10.3389/fimmu.2020.00724 32391019 PMC7190872

[B38] Yin X. Zhao C. Qiu Y. Zhou Z. Bao J. Qian W. (2021). Dendritic/Post-synaptic Tau and early pathology of Alzheimer’s disease. *Front. Mol. Neurosci.* 14:671779. 10.3389/fnmol.2021.671779 34248498 PMC8270001

[B39] Zhang C. Zhao M. Wang B. Su Z. Guo B. Qin L. (2021). The Nrf2-NLRP3-caspase-1 axis mediates the neuroprotective effects of Celastrol in Parkinson’s disease. *Redox Biol.* 47 102134. 10.1016/j.redox.2021.102134 34600334 PMC8487081

[B40] Zhang H. Hong Y. Yang W. Wang R. Yao T. Wang J. (2021). SNX14 deficiency-induced defective axonal mitochondrial transport in Purkinje cells underlies cerebellar ataxia and can be reversed by valproate. *Natl. Sci. Rev.* 8:nwab024. 10.1093/nsr/nwab024 34691693 PMC8310771

